# 造血干细胞移植相关克隆性造血研究进展

**DOI:** 10.3760/cma.j.cn121090-20250209-00060

**Published:** 2026-01

**Authors:** 舟 金, 艺尹 陈, 蒙 单, 杨 徐

**Affiliations:** 苏州大学附属第一医院，国家血液系统疾病临床医学研究中心，江苏省血液研究所，卫生部血栓与止血重点实验室，苏州大学造血干细胞移植研究所，苏州 215006 The First Affiliated Hospital of Soochow University, National Clinical Research Center for Hematologic Diseases, Jiangsu Institute of Hematology, Key Laboratory of Thrombosis and Hemostasis Under Ministry of Health, Institute of Blood and Marrow Transplantation, Soochow University, Suzhou 215006, China

## Abstract

随着高通量测序技术的发展，克隆性造血（CH）在心脑血管疾病及血液系统疾病领域的研究中受到广泛关注。造血干细胞移植（HSCT）作为治疗恶性血液系统疾病的有效手段，尽管显著提高了患者的生存率，但移植后移植物抗宿主病、治疗相关髓系恶性肿瘤及疾病复发等不良预后问题的确切机制尚未完全阐明。近年来，研究表明CH相关的突变基因（如DNMT3A、PPM1D等）可能与HSCT的不良预后密切相关。本文就HSCT相关CH的遗传因素、诱因、研究进展及其对患者预后的影响进行系统综述，为进一步探索相关机制及优化HSCT治疗方案提供理论基础。

## Abstract (en)

With the development of high-throughput sequencing technology, clonal hematopoiesis has received extensive attention in the research of cardiovascular, cerebrovascular, and hematological diseases. Hematopoietic stem cell transplantation（HSCT）is an effective treatment for malignant hematological diseases and has considerably improved the survival rate of patients. However, the exact mechanisms underlying the adverse outcomes, such as graft-versus-host disease, treatment-related myeloid neoplasms, and disease recurrence after transplantation, have not been fully elucidated. Recent studies have shown that mutation genes related to clonal hematopoiesis（such as DNMT3A and PPM1D）may be closely related to the adverse prognosis of HSCT. The present article systematically summarizes the genetic factors, inducements, research progress, and impact on patient prognosis of HSCT-related clonal hematopoiesis, providing a theoretical basis for further exploration of the related mechanisms and optimization of HSCT regimens.

克隆性造血（CH）是指在骨髓造血干细胞（HSC）和祖细胞中，衰老相关的驱动性突变形成的体细胞嵌合体，导致HSC的生存和生长受到影响的病理状态[1]。驱动突变包括血液肿瘤相关基因突变、镶嵌体染色体改变以及性染色体丢失等；不明的驱动突变导致的克隆异常在排除不明原因血细胞减少症后，即可定义为具有不确定潜力的克隆性造血（CHIP）[1]–[3]。研究表明，CH的发生率随年龄增长显著上升：40岁以下人群CH发生率较低，50岁人群为1％～2％，而65岁人群可达5％～20％。此外，男性、吸烟以及西班牙裔等特征人群的CHIP发生率较高[4]–[5]。CH的存在与血液系统恶性肿瘤发生风险增加、全因死亡率升高以及冠心病和缺血性卒中的发生密切相关[1]。

造血干细胞移植（HSCT）是恶性血液病的重要治疗手段。然而，若发生基因突变、染色体易位等遗传学异常事件，可能导致干祖细胞分化受阻或增殖异常，进而影响HSCT疗效。随着降低强度预处理方案的推广，目前研究表明，CH在接受HSCT的患者中的检出率高于普通人群的一般水平（5％～20％）；自体造血干细胞移植（auto-HSCT）患者的CH中位患病率为26.7％，而异基因造血干细胞移植（allo-HSCT）中，供者和患者的CH中位患病率分别达23％和31％[6]。老年患者是CH的高发人群，且降低强度预处理方案的推广使得更多老年患者接受HSCT，尤其以兄弟姐妹作为主要供体来源，进一步导致移植相关人群中CH携带者比例上升。此外，高通量测序技术的发展为揭示CH对HSCT预后的影响提供了新的研究契机。本文就HSCT相关CH的遗传因素、诱因、研究进展及其对预后的影响作一综述。

一、HSCT相关CH的突变基因

Steensma等[7]推荐以下基因可作为CHIP诊断依据：DNA甲基转移酶3A（DNMT3A）、TET甲基胞嘧啶双加氧酶2（TET2）、酪氨酸蛋白激酶2（JAK2）、RNA剪接因子3B亚基1（SF3B1）、附加性别梳样蛋白1（ASXL1）、TP53、E3泛素-蛋白连接酶CBL（CBL）、鸟嘌呤核苷酸结合蛋白亚基β1（GNB1）、BCL6共抑制因子（BCOR）、剪接因子U2AF1（U2AF1）、组蛋白乙酰转移酶CREB结合蛋白（CREBBP）、同源盒蛋白Cux-1（CUX1）、丝氨酸/精氨酸富集剪接因子2（SRSF2）、组蛋白-赖氨酸N-甲基转移酶2D（KMT2D）、组蛋白-赖氨酸N-甲基转移酶SETD2（SETD2）、组蛋白-赖氨酸N-甲基转移酶SETDB1（SETDB1）、鸟嘌呤核苷酸结合蛋白亚基α（GNAS）、金属依赖性蛋白磷酸酶1D（PPM1D）、BCL6共抑制因子样1（BCORL1），并建议以变异等位基因频率（VAF）≥2％为判定标准。但是CH的循证VAF标准尚未明确[8]。HSCT相关CH主要通过2种途径影响移植疗效：①在allo-HSCT中，供者来源的CH相关基因突变随移植物输入患者，进而影响移植预后；②在auto-HSCT中，患者自身存在的CH突变可直接影响移植结局。目前研究发现，HSCT-CH相关基因主要涉及两大功能类别：一类是表观遗传调控相关基因，包括DNMT3A、ASXL1、TET2；另一类是DNA损伤修复相关基因，如PPM1D、TP53。其中，DNMT3A和PPM1D突变率较高，且具有显著的移植类型特异性：DNMT3A突变在allo-HSCT中更为常见[9]；而PPM1D突变则在auto-HSCT中的发生率高[10]。

1. DNMT3A：DNMT3A是重要的表观遗传修饰基因，也是CH中最常见的突变基因，约50％的CH相关突变发生于该基因[11]。在allo-HSCT中，供者DNMT3A突变发生率为10％～20％，而患者的突变发生率则为30％～50％[9],[12]–[13]。DNMT3A通过甲基化调控造血细胞增殖分化，当其发生突变时，易导致CH的发生。一项基于小鼠模型的研究揭示了DNMT3A突变在促进CH中的作用[11]，研究发现，DNMT3A^−/−^HSC小鼠表现出增强的自我更新和降低的分化效率，导致表型正常的HSC异常积累。在髓系疾病患者中，DNMT3A突变主要集中在R882位点。DNMT3A^R882^具有显性负调控功能，抑制野生型DNMT3A的甲基转移酶活性，从而促进细胞自我更新，抑制其分化功能[11]。此外，研究显示在携带DNMT3A^R882^突变的急性髓系白血病（AML）患者中，该突变常与核仁磷酸蛋白1（NPM1）和FMS样酪氨酸激酶3基因内部串联重复突变序列（FLT3^ITD^）突变共存，并对患者的完全缓解（CR）率与总生存（OS）期产生负面影响[14]。

2. TET2：TET2突变在HSCT相关CH中的发生率为10％～30％[9],[15]。作为表观遗传调节因子，TET2通过调节DNA甲基化和大量基因的表达，例如肾母细胞瘤1（WT1）、FLT3，以维持细胞特性并抑制肿瘤发生[16]，其突变最常发生于3号和11号外显子[17]。TET2和DNMT3A共同修饰CpG二核苷酸上的胞嘧啶，不同于DNMT3A仅添加甲基，TET2则是将5-甲基胞嘧啶修饰为5-羟甲基胞嘧啶[18]–[19]。目前研究表明，TET2突变具有显著的致病性和临床侵袭性，可加速疾病进展并导致患者预后不良[20]。例如，在慢性粒-单核细胞白血病（CMML）中，诊断时存在CCAAT增强子结合蛋白α（CEBPA）突变且并发双等位基因TET2突变与神经母细胞瘤RAS病毒癌基因同源物（NRAS）突变是促进AML转化和缩短OS期的独立危险因素[21]。有研究测试TET2基因敲除的HSC在小鼠体内移植环境中直接与野生型细胞竞争，结果证实TET2缺失与HSC自我更新增加，分化停滞，粒、单核细胞偏倚和髓系转化增加相关[22]。

3. ASXL1：染色质修饰基因ASXL1位于染色体20q11，属于trithorax家族（TrxG）和polycomb家族（PcG）的增强子，其体细胞突变位点大多位于第12号外显子[23]。ASXL1通过影响组蛋白修饰，在调节核激素受体的转录活性中发挥激活或抑制作用，例如激活视黄酸受体介导的转录过程，抑制过氧化物酶体增殖物激活受体α（PPARα）和肝X受体α（LXRα）介导的转录过程[24]。在HSCT相关CH中，ASXL1突变的发生率为10％～20％，其与DNMT3A、TET2基因构成的DTA基因突变组对HSCT患者预后具有一定的影响[25]。在骨髓微环境中，ASXL1对正常造血至关重要，而骨髓间充质干细胞（BMSC）中的ASXL1缺失会改变HSC/造血祖细胞池，导致髓系分化偏倚[26]。

4. PPM1D：PPM1D为DNA损伤反应（DDR）的负调节因子，其突变在各年龄段中发生率较低，为0.13％～0.16％，但是PPM1D突变在经历过化疗的患者中发生率较高，为3.4％～23.5％[27]，这可能与PPM1D突变对细胞毒性暴露的耐受性较强有关。因此，在接受过大剂量化疗的auto-HSCT患者中，PPM1D突变发生率一般高于10％[28]–[29]。PPM1D突变通常发生在末端6号外显子，并由p53诱导表达。在接受移植前化疗预处理中，DDR激活了细胞保护机制，发挥抗增殖和肿瘤抑制作用。然而，PPM1D突变通过抑制DDR通路的关键组分的活性，包括p53、共济失调毛细血管扩张症突变激酶（ATM）、细胞周期检测点激酶1/2（CHK1/2）或p38-丝裂原活化蛋白激酶（p38-MAPK）[30]，从而抑制由DDR介导的细胞凋亡，进而促进HSC克隆性增殖。

5. 其他基因：除上述基因以外，根据2022年世界卫生组织提出的造血淋巴肿瘤分型诊断标准，JAK2、TP53、SF3B1、SRSF2、异柠檬酸脱氢酶1/2（IDH1/2）、U2AF1、KRAS/NRAS、CCCTC结合因子（CTCF）、CBL、GNB1、BRCA1-BRCA2复合物亚基3（BRCC3）、蛋白酪氨酸磷酸酶非受体11型（PTPN11）、GNAS、BCORL1等也是CHIP的必检基因，这些基因在不同血液疾病中具有独特的预后意义。例如，在CMML中，剪接体基因SF3B1、SRSF2、U2AF1突变驱动疾病进展[31]；在骨髓增生异常肿瘤（MDS）中，DTA共突变联合IDH2、BCOR或CBL突变提示不良预后[32]。未来研究需进一步探讨这些基因在HSCT相关CH预后模型中的具体作用机制及其临床意义。

二、HSCT相关CH的诱因

CH自胚胎期起就在人体内出现，主要由基因突变和染色体畸变引起。除了受到生活习惯、地域环境、理化刺激[33]等外源性因素的影响，CH突变通常在接受HSCT的患者中扩展，并可能引发新的、更具侵袭性的促癌突变[6]。随着年龄的增长，诸如炎症反应、骨髓微环境衰老、代谢压力及免疫紊乱等因素可能促使CH进一步发展。此外，HSCT相关CH还受到移植前基础疾病及预处理等特定因素的影响。

1. 移植引发的炎症反应：移植引发的全身性炎症反应贯穿CH发生与演进的全过程[34]。在移植过程中，受体细胞及其所处的骨髓微环境对供体细胞植入所产生的应激反应，可能引发体内炎症因子水平升高，继而刺激CH的发生发展。在此背景下，HSC的加速循环和自我更新提高了DNA复制过程中突变的发生概率，基因突变的HSC克隆进一步促进炎症反应的持续加剧[35]。例如，γ干扰素（IFN-γ）介导的炎症反应可促进DNMT3A突变的HSC扩增[36]，进而导致植入供体DNMT3A突变HSC的患者体内IL-12p70水平显著高于其他受体（共241例，0.37 pg/ml对0.16 pg/ml，*P*＝0.002）[8]。由此提示，供者产生的CH可能通过与患者体内由移植引起的高水平炎症细胞因子的相互作用，进一步加速CH的进展。

2. 移植相关化疗及预处理：移植过程中使用的化疗药物及移植前的预处理可能加剧特定的CH基因突变，白消安、环磷酰胺、羟基脲、甲氨蝶呤等药物已被证实能引起CH基因突变[37]。作为一种外在压力，移植前的预处理同时刺激了供受体细胞及骨髓微环境，从而为CH的发生和进展提供有利条件[38]。一项研究提示75例多发性骨髓瘤患者在第1次或第2次高剂量环磷酰胺治疗和auto-HSCT后，缓解的外周血细胞中存在PPM1D突变[34]。同样，另一项研究对401例接受auto-HSCT的非霍奇金淋巴瘤患者进行CH分析，结果表明接受大剂量清髓性化疗的患者，其移植后PPM1D和TP53突变的频率显著高于一般人群，其中PPM1D突变频率较普通人群高出75至92倍；而TP53突变率在此研究中也高达0.5％，远超其在普通人群中0.005％至0.02％的水平[39]。进一步的研究在119例淋巴瘤和骨髓瘤患者中对驱动基因克隆进行分析，发现与未接受化疗的患者相比，移植前接受大剂量化疗的auto-HSCT患者的CH突变量更高[34]，提示移植前预处理在CH进展中起到关键作用。

3. 移植前基础疾病：患者移植前的基础血液疾病特征驱动特定的基因突变谱，进而塑造诊断时可检出的CH相关突变。例如，根据欧洲白血病网（ELN）预后分层系统，预后较差的AML中常见ASXL1、TP53、SF3B1和BCOR等基因突变；骨髓纤维化患者通常携带JAK2和钙网蛋白突变。这些疾病特异的突变谱不仅反映了基础病理状态，还通过影响克隆演化轨迹和微环境重塑，对移植治疗效果产生显著影响。以AML为例，因基础病理状态而存在的DTA突变可在诱导化疗及移植后持续残留，可能导致不良的移植预后[40]。此外，其他移植前基础疾病如心血管疾病、慢性肝病和肺部感染也可能对CH进展产生一定影响[6]，但其与CH进展的因果关系仍缺乏深入研究，未来需要更多的数据加以阐明。

4. 衰老的骨髓微环境：移植时供体年龄的增加可能与HSCT相关CH的风险增加有关[41]–[42]，提示衰老的骨髓微环境可能使CH更易发生。例如，衰老的骨髓微环境中的BMSC损害造血干祖细胞的克隆形成潜力并诱导产生促炎因子以促进CH细胞的产生及增殖[43]–[44]。与此同时，CH细胞又作用于微环境，使其加速衰老更利于自身的存活和进展，例如DNMT3A^R878H/+^造血干祖细胞导致MSC产生的衰老标志物SA-β-gal、Bcl-2、Bcl-xL、Cdkn1a（p21）和Cdkn2a（p16）水平升高[44]，因此衰老的微环境是患者移植后体内CH的重要危险因素。骨髓间充质细胞在衰老骨髓微环境中的作用已得到广泛关注[43]–[44]，研究表明，衰老的间充质基质细胞通过细胞因子和信号通路的改变，可能在驱动CH发生中起着关键作用。与此同时，衰老骨髓微环境中炎症因子如TNF和IFN-γ的升高也被认为可以推动CH的发生与进展[45]–[46]，研究发现与年龄相关的TNF增加可促进老年受体小鼠中TET2^−/−^细胞的扩增，且这一扩增倾向于髓系分化。靶向TNF可能为缓解某些形式的CHIP负担提供潜在的治疗策略[47]。此外，接受移植的老年患者骨髓微环境在维持代谢稳定性方面的能力下降，可能与活性氧水平升高[48]及线粒体腺苷三磷酸（ATP）生成增加密切相关。

5. 免疫紊乱：绝大多数CH不会直接导致血液恶性肿瘤的发生，免疫系统在其发生和进展中仍然发挥着一定的抑制作用[49]–[50]。然而，CH的存在本身证明，携带突变的造血干细胞/祖细胞已成功克服免疫监视，实现了克隆性扩增。HSCT过程中由炎症因子[50]、免疫细胞衰老[49]等因素引发的免疫失调，为免疫逃逸提供了有利条件。免疫调节药物有助于减轻接受移植的患者体内CH的负面影响，提示免疫失调与CH的发生和进展密切相关。例如，一项研究发现，VAF≥1％的DNMT3A突变驱动的克隆性造血（DNMT3A-CH）与未接受移植后环磷酰胺（PTCy）治疗的患者慢性GVHD的发生风险增加独立相关［亚分布风险比（s*HR*）＝1.37，95％ *CI*：1.02～1.84；*P*＝0.04］，然而对于接受PTCy治疗的患者，DNMT3A-CH对慢性GVHD的发生并无影响（s*HR*＝1.15，95％ *CI*：0.82～1.60；*P*＝0.88）[8]。此外，在血液系统恶性肿瘤背景下，骨髓微环境的改变亦有助于CH克隆的免疫逃逸，其机制可能涉及突变HSC通过表达特定免疫调节基因，主动适应并抵抗机体的免疫监视压力，从而实现在免疫监视下的优势存活与扩增[51]。

三、CH对HSCT的预后影响

CH对allo-HSCT以及auto-HSCT的临床影响各异：前者主要表现为GVHD风险增高，后者则主要驱动治疗相关髓系肿瘤（T-MN）发生，为auto-HSCT患者生存率下降及复发增加提供了部分解释。鉴于CH对两类移植结局的明确影响，在移植前评估中纳入供者与患者的CH状态具有一定的临床价值。2019年到2024年供患者CH相关临床研究报道总结于表1。

**表1:** 2019年至2024年造血干细胞移植相关克隆性造血（CH）的临床研究报道

文献来源	移植方式	CH来源	检测时间	检测CH样本量（例/名）	移植前基础血液疾病	主要相关基因	CH判定VAF阈值	CH对患者移植结局的影响（CH组对非CH组）	
								移植后疾病状态	移植并发症及其他重要事件
Frick等[9]	allo-HSCT	供者	移植前	500	AML、MDS、MPN、ALL、CLL	DNMT3A、TET2、ASXL1	≥2.00％	疾病复发率降低（*HR*＝0.63，95％ *CI*：0.41～0.98，*P*＝0.042）	cGVHD发生风险升高（*HR*＝1.73，95％ *CI*：1.21～2.49，*P*＝0.003）
Kim等[12]	allo-HSCT	供者	移植前	744	AA、AML、MDS、MPN、ALL、CLL、NHL、CML、MM	TET2、DNMT3A、SMC3、SF3B1	≥0.62％	–	–
Oran等[13]	allo-HSCT	供者	移植前	363	AML、MDS	DNMT3A、TET2、ASXL1、PPM1D	≥2.00％	–	Ⅱ～Ⅳ级（*HR*＝2.4，95％ *CI*：1.6～3.6，*P*<0.001）和Ⅲ～Ⅳ级（*HR*＝3.8，95％ *CI*：1.6～8.9，*P*＝0.003）aGVHD发生风险升高
Lackraj等[28]	allo-HSCT	患者	移植后	181	DLBCL、MCL、HL、TIL、TCL	PPM1D、TET2、DNMT3A、ASXL1、TP53	≥0.10％	OS期缩短（*HR*＝1.46，95％ *CI*：1.06～2.01，*P*＝0.020）；造血重建延迟：中性粒细胞（*HR*＝1.25，95％ *CI*：1.01～1.56，*P*＝0.045）和血小板（*HR*＝1.54，95％ *CI*：1.22～1.95，*P*<0.001）恢复时间延长	–
Husby等[29]	auto-HSCT	患者	移植后	440	HL、NHL	PPM1D、TP53、RAD21、BRCC3	≥2.00％	OS期（*HR*＝2.79，95％ *CI*：1.71～4.56，*P*<0.001）和EFS期（*HR*＝2.69，95％ *CI*：1.61～4.49，*P*<0.001）缩短	T-MN发生风险升高（*HR*＝6.50，95％ *CI*：2.34～18.03，*P*<0.001）
Wei等[52]	allo-HSCT	患者	移植前	196	AML	DNMT3A、TET2、IDH1、IDH2、ASXL1	–	–	aGVHD发生风险升高：在年龄≥45岁患者中，CH组Ⅱ～Ⅳ级aGVHD发生率显著高于非CH组（69.0％对45.0％，*P*＝0.048）
Awada等[53]	auto-HSCT	患者	移植后	1 507	MM、NHL、HL	PPM1D、TP53	≥2.00％	CH组T-MN患者中位OS期缩短（9.8个月对23.8个月，*P*＝0.03），1年死亡率更高（66.7％对20％，*P*＝0.01）	T-MN发生风险显著升高：CH组（PPM1D或TP53突变）患者中T-MN发生率高于非CH组（PPM1D突变：12.9％对2.0％，*P*＝0.05；TP53突变：9.7％对0，*P*＝0.02）
Ortmann等[54]	allo-HSCT	患者	移植后	81	MM、HL	DNMT3A、TET2、TP53、PPM1D、RAD21	≥0.50％	造血重建延迟：CH是中性粒细胞重建延迟的独立危险因素（*HR*＝2.77，*P*＝0.002，原文未报告置信区间）	–
Rhee等[55]	auto-HSCT	患者	移植前	1 036	MM	DNMT3A、ASXL1、TET2、TP53	≥2.00％	–	心血管疾病发生风险升高（5年发生率21.1％对8.4％，*P*<0.001）

**注** VAF：变异等位基因频率；allo-HSCT：异基因造血干细胞移植；AML：急性髓系白血病；MDS：骨髓增生异常肿瘤；MPN：骨髓增殖性肿瘤；ALL：急性淋巴细胞白血病；CLL：慢性淋巴细胞白血病；DNMT3A：DNA甲基转移酶3A；TET2：TET甲基胞嘧啶双加氧酶2；ASXL1：附加性别梳样蛋白1；cGVHD：慢性移植物抗宿主病；AA：再生障碍性贫血；NHL：非霍奇金淋巴瘤；CML：慢性髓细胞性白血病；MM：多发性骨髓瘤；SMC3：SMC3黏连蛋白复合体成分；SF3B1：RNA剪接因子3B亚基1；PPM1D：金属离子依赖性蛋白磷酸酶1D；aGVHD：急性移植物抗宿主病；auto-HSCT：自体造血干细胞移植；DLBCL：弥漫大B细胞淋巴瘤；MCL：套细胞淋巴瘤；HL：霍奇金淋巴瘤；TIL：转化性惰性淋巴瘤；TCL：T细胞淋巴瘤；TP53：肿瘤蛋白P53；OS：总生存；RAD21：RAD21黏连蛋白复合体成分；BRCC3：BRCA1-BRCA2复合物亚基3；EFS：无事件生存；T-MN：治疗相关髓系肿瘤；IDH1：异柠檬酸脱氢酶1；IDH2：异柠檬酸脱氢酶2；–：未提及

1. allo-HSCT供者来源CH对移植疗效的影响：

（1）GVHD：HSCT相关CH对GVHD的影响仍存在一定的争议。Oran等[13]通过对300例MDS/AML的队列研究发现，供体存在CH的患者移植后6个月发生Ⅱ～Ⅳ级（*HR*＝2.4，95％ *CI*：1.6～3.6，*P*<0.001）和Ⅲ～Ⅳ级（*HR*＝3.8，95％ *CI*：1.6～8.9，*P*＝0.003）急性GVHD的风险更高。而另一项研究表明供者CH与慢性GVHD的发生呈显著相关（s*HR*＝1.36，95％ *CI*：1.01～1.83，*P*＝0.042）[8]。以上研究支持CH可能通过增加GVHD的发生风险对allo-HSCT的临床预后产生影响。然而，Kim等[12]进行的一项长达13年的随访研究则发现CH对GVHD并无显著影响。因此，HSCT相关CH对GVHD的影响尚需进一步深入研究。

（2）生存及复发：现有临床研究表明，供者CH（DNMT3A R882^mut^/FLT3-ITD^pos^）与移植后复发率降低相关，这可能是由于供者DNMT3A突变细胞与患者体内残存的肿瘤细胞之间发生竞争，供者突变细胞因其生长优势而抑制了残留肿瘤细胞的增殖，从而降低复发风险[9]。然而，目前尚无关于CH对总复发死亡率、非复发死亡率及无进展生存期影响的统一结论[8]–[9],[13]。一项回顾性研究提示持续存在于AML CR患者体内的DNMT3A、ASXL1、TET2突变与更差的生存结局相关，具体表现为更高的复发率及更短的无病生存期和OS期[56]。关于供者CH对患者复发及生存的影响，仍需更多的队列研究加以验证。

（3）其他预后影响：多数研究提示，CH与allo-HSCT的植入及造血重建之间并未发现相关性。然而，也有少数回顾性研究提示，CH与血小板及中性粒细胞的重建延迟相关[28],[54]。供体来源的CH是移植后发生供体细胞白血病的重要风险因素[57]，但其发生率较低，且样本量有限，未来仍需要进一步研究来明确其临床意义。

2. allo-HSCT患者CH相关基因突变对移植疗效的影响：患者在移植前已存在CH相关基因突变时，接受正常供者干细胞移植后，其疗效可能受到CH的复杂影响。一方面，供者干细胞可能通过竞争性造血抑制患者CH，避免CH对移植疗效产生不良影响，如一项研究显示在22例接受年轻供者HSCT治疗8年后的老年患者中，只有1例检测出CH相关基因突变[58]，其移植后心血管疾病的发生风险高，而其余患者均未受CH对移植疗效的不良影响。另一方面，患者移植前已存在的CH在移植后可能持续存在，即可测量的残余克隆造血（MRCH），并与不良预后密切相关，包括OS期与无事件生存（EFS）期缩短，复发及GVHD风险增加[15],[52]，如一项由Bischof等[15]进行的研究通过动态监测证实，移植后检测到的MACH是移植后长期EFS的显著且持续的风险预测因子。其风险比（*HR*）在移植后多个时间点均具有关联性，包括移植后28 d（*HR*＝6.25，95％ *CI*：2.72～14.58，*P*＝0.002）、100 d（*HR*＝4.49，95％ *CI*：1.59～12.67，*P*<0.001）及180 d（*HR*＝6.36，95％ *CI*：1.41～28.54，*P*＝0.003）[15]。另外，移植后出现的MACH在预测疾病复发方面展现出卓越价值，其预测效能与微小残留病（MRD）相当：移植后28 d［曲线下面积（AUC）_MACH_＝0.98对AUC_MRD_＝0.94，*P*＝0.69］、56 d（AUC_MACH_＝0.89对AUC_MRD_＝0.92，*P*＝0.52）及84 d（AUC_MACH_＝0.83对AUC_MRD_＝0.75，*P*＝0.49）；AUC_MACH_在移植后28 d达到最高，这一结果提示在风险预警方面，CH具有作为新型动态监测标志物的潜力[15]。

因此，患者CH相关基因突变对移植疗效的影响既可能受供者HSC状态的影响，也受自身克隆演化动态的影响。当前研究存在方法学局限性，因多数研究仅对供者或患者单方面进行CH基因突变检测，缺乏同步动态监测。这种单维度的分析模式难以全面解析供受者CH的交互作用，可能导致预后评估偏差。未来需通过纵向追踪供受者双方的CH克隆演变以便更准确地界定其对移植结局的影响。

3. auto-HSCT相关CH与T-MN的关联：T-MN是霍奇金淋巴瘤（HL）患者接受auto-HSCT后可能出现的严重并发症[53],[59]。在auto-HSCT中，CH对T-MN的影响已逐步得到验证。Yan等[59]关于321例HL患者的回顾性队列研究表明，外周血干细胞中存在CH的患者，T-MN的发生风险增高（a*HR*＝4.50，95％ *CI*：1.54～13.19，*P*＝0.0061）。此外，TP53和（或）PPM1D突变的存在与T-MN的发生风险相关（*HR*＝7.29，95％ *CI*：1.72～30.94，*P*＝0.0071），且这些突变与非复发死亡率的升高密切相关（*HR*＝4.17，95％ *CI*：1.25～13.87，*P*＝0.020）[59]。由此可见，CH突变与T-MN的发生存在较强的关联，未来可能通过抑制相关基因的功能，开发预防和治疗T-MN的靶向策略[9],[12],[27]。

四、总结

CH在HSCT中可能作为预后评估的潜在生物标志物，特别是对于GVHD、T-MN、生存及复发的影响。然而，目前关于CH在HSCT中的研究大多集中于中老年患者，针对40岁以下年轻供患者的相关研究较少。随着高通量测序技术的发展，未来可进一步探讨CH基因演化的规律，并加深对HSCT治疗过程的理解。对HSCT中CH及其具体模式的深入研究，有助于揭示供患者细胞的生物学特性，并从危险分层、治疗时机和策略等多个维度优化诊疗方案，从而在供患者选择、预后监测及个性化管理等方面实现更高水平的疗效。
